# Differential expression of three galaxin-related genes during settlement and metamorphosis in the scleractinian coral *Acropora millepora*

**DOI:** 10.1186/1471-2148-9-178

**Published:** 2009-07-29

**Authors:** Alejandro Reyes-Bermudez, Zhiyi Lin, David C Hayward, David J Miller, Eldon E Ball

**Affiliations:** 1 ARC Centre of Excellence for Coral Reef Studies and Comparative Genomics Centre, James Cook University, Townsville, Qld, 4811, Australia; 2 Centre for Molecular Genetics of Development and Research School of Biological Sciences, Australian National University, PO Box 475, Canberra, ACT, 2601, Australia

## Abstract

**Background:**

The coral skeleton consists of CaCO_3 _deposited upon an organic matrix primarily as aragonite. Currently galaxin, from *Galaxea fascicularis*, is the only soluble protein component of the organic matrix that has been characterized from a coral. Three genes related to *galaxin *were identified in the coral *Acropora millepora*.

**Results:**

One of the *Acropora *genes (*Amgalaxin*) encodes a clear galaxin ortholog, while the others (*Amgalaxin-like 1 *and *Amgalaxin-like 2*) encode larger and more divergent proteins. All three proteins are predicted to be extracellular and share common structural features, most notably the presence of repetitive motifs containing dicysteine residues. In situ hybridization reveals distinct, but partially overlapping, spatial expression of the genes in patterns consistent with distinct roles in calcification. Both of the *Amgalaxin-like *genes are expressed exclusively in the early stages of calcification, while *Amgalaxin *continues to be expressed in the adult, consistent with the situation in the coral *Galaxea*.

**Conclusion:**

Comparisons with molluscs suggest functional convergence in the two groups; lustrin A/pearlin proteins may be the mollusc counterparts of galaxin, whereas the galaxin-like proteins combine characteristics of two distinct proteins involved in mollusc calcification. Database searches indicate that, although sequences with high similarity to the galaxins are restricted to the Scleractinia, more divergent members of this protein family are present in other cnidarians and some other metazoans. We suggest that ancestral galaxins may have been secondarily recruited to roles in calcification in the Triassic, when the Scleractinia first appeared. Understanding the evolution of the broader galaxin family will require wider sampling and expression analysis in a range of cnidarians and other animals.

## Background

Although calcification – the deposition of calcium salts, usually to provide a form of skeletal support – is a widespread trait among animals, scleractinian corals are distinguished by the scale on which they carry out this process. In contrast to the internal skeletons of tetrapods and bony fish, which are based on calcium phosphate in the form of hydroxyapatite, many invertebrate skeletons are composed of CaCO_3_. This may take the form of either calcite or aragonite, which have distinct structural properties. Both forms are known in cnidarians and molluscs, with the massive exoskeletons of extant scleractinian corals consisting predominantly of the latter.

Scleractinians are responsible for the underlying framework of coral reefs and are the most obvious calcifying animals in warm shallow waters of the tropics and sub-tropics. Calcification by corals is known to be strongly enhanced by light (e.g. [1,2]) and is therefore assumed to be driven indirectly by photosynthesis in their dinoflagellate symbionts, but very little is known of the mechanism of calcification or the nature of the organic matrix on which the CaCO_3 _of the skeleton is deposited.

The difference in the type of CaCO_3 _polymorph used to build the wide range of invertebrate support structures reflects the biological control exerted by the organism during mineral deposition. This is achieved via a macromolecular network of proteins, lipids and polysaccharides [3-5] known as the skeletal organic matrix (SOM). Prior to mineralization, this matrix is secreted to the extracellular space by the calcifying cells and there induces CaCO_3 _nucleation, determining the type of polymorph as well as size and shape of crystals [6-11]. Although the process of calcification and the nature of the calcifying matrix have been extensively studied in molluscs [12-14], there have been relatively few corresponding studies on corals. To date, the only protein to have been fully characterised from the calcifying matrix of scleractinian corals is galaxin, which was originally identified from the coral *Galaxea fascicularis *[8].

**Figure 1:**
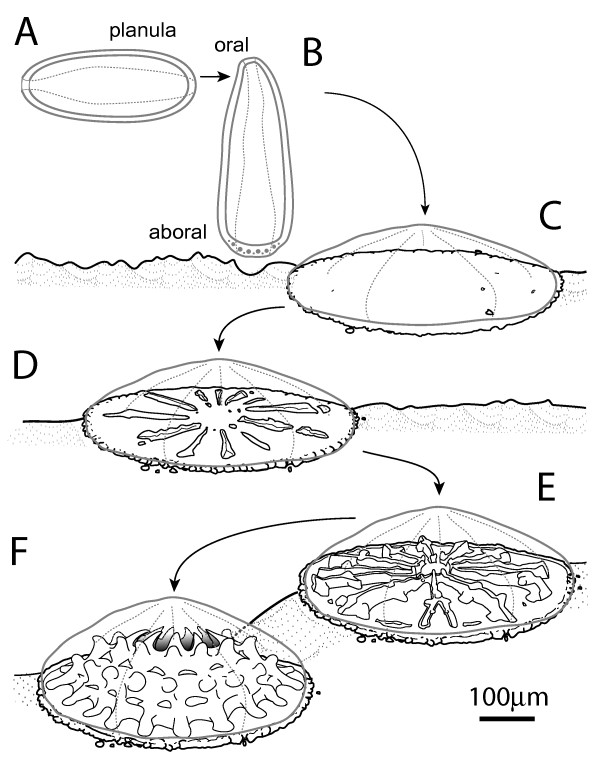
**Diagrammatic summary of the morphological changes involved in *Acropora *settlement, metamorphosis and the initiation of calcification**. (A) Initially the planula swims horizontally, well away from the bottom. (B) After it has become competent to settle it explores the substratum with its aboral end. (C) If it encounters appropriate chemical cues it rounds up into a sphere, which then flattens against the substratum. As this is happening the cells against the substratum change their morphology and begin to secrete a disc of organic material. (D) Perpendicular walls, the protosepta, are then secreted on the plate in multiples of six. (E) The protosepta become more elaborate and more are added. (F) The walls become joined laterally and grow upward, forming a crown shaped structure upon which the polyp sits. In all stages the skeleton is external to, but enveloped by, the living tissue which secretes it. By the stages shown in (E) and (F) a polyp has formed, initially with tentacle buds and then with tentacles. However, these have been omitted from the drawing in order to focus on the skeleton. The drawings are based on drawings in Goreau and Hayes [34].

Although calcification is initiated immediately after settlement (Figure 1; [15]), most studies have focussed on soluble components of the organic matrix of adult coral skeletons. The presence of the massive skeleton, however, complicates analysis of the process of calcification, whereas the period immediately following settlement is likely to be more tractable. The initiation of larval skeleton deposition correlates with the aboral ectodermal transition that gives rise to the calicoblastic ectoderm [15-18]. Larval skeleton deposition does not require the presence of zooxanthellae, but generates a template for the skeleton of the initial polyp [3,15,16]. Both the organization and orientation of calcium carbonate differs between larval and adult calcification; larval skeletons are more lightly mineralised and may contain amorphous calcium carbonate, calcite and aragonite crystals. When the latter are present they are in the form of smaller crystals that are randomly orientated whereas adult skeletons are exclusively composed of aragonite crystals organized in parallel fibers [19]. It is unclear whether this apparent difference between larval and adult calcification patterns represents two distinct types of calcification, or ontogenetic stages in the development of a largely aragonite skeleton as is the case in molluscs [20]. In either case, the immediate post-settlement period is the only time during the life cycle of the coral that calcification, and the role of the organic matrix in that process, can be studied in isolation and it thus presents a unique opportunity to investigate the molecular bases of coral calcification.

An ongoing EST project on the staghorn coral *Acropora millepora *[21,22] has allowed the identification of genes encoding candidate calcifying matrix components. Here we report the sequences of three genes encoding galaxin-related molecules in *Acropora *and their expression patterns during settlement and metamorphosis. Based on these data, we suggest distinct roles for the three gene products during the two characteristic phases of calcification observed during the coral life cycle. This paper is particularly topical given the current concern over the effects of rising levels of atmospheric CO_2 _on the acidity of the oceans, and consequently on the ability of marine organisms to lay down their CaCO_3 _skeletons.

## Results

### Identification of three *galaxin*-related genes in *Acropora*

Two unigenes identified during EST analyses of *Acropora millepora *gave strong matches with *Gfgalaxin*, the gene coding for a soluble protein identified as a major component of the exoskeleton of the coral *Galaxea fascicularis *[8,11]. One of these (*Amgalaxin*) codes for a protein assumed to be the *Acropora *ortholog of Gfgalaxin, as the predicted proteins had 58% identity and 73% similarity overall (BlastP significance = 1e^-105^). The second unigene, *Amgalaxin-like 1*, encodes a larger protein with much lower overall identity to Gfgalaxin (BlastP significance = 7e^-19^). An additional related unigene, *Amgalaxin-like 2*, was identified in a screen for genes differentially regulated at the time of settlement, and has a BlastP significance against Gfgalaxin of 2e^-33^.

### Structure of the predicted galaxin-related proteins from *Acropora*

**Figure 2:**
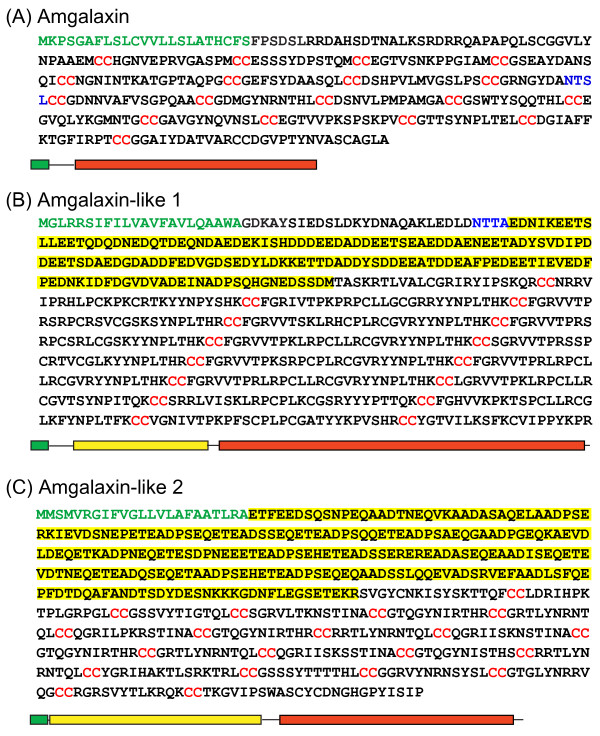
**Inferred protein sequences of Amgalaxin and the Amgalaxin-like molecules**. Amgalaxin differs from the galaxin-like molecules in that it lacks an acidic domain. The signal peptide is marked in green, the acidic domain in yellow, and the di-Cys repeats in red. Potential N-linked glycosylation sites in Amgalaxin and Amgalaxin-like-1 are shown in blue.

**Figure 3:**
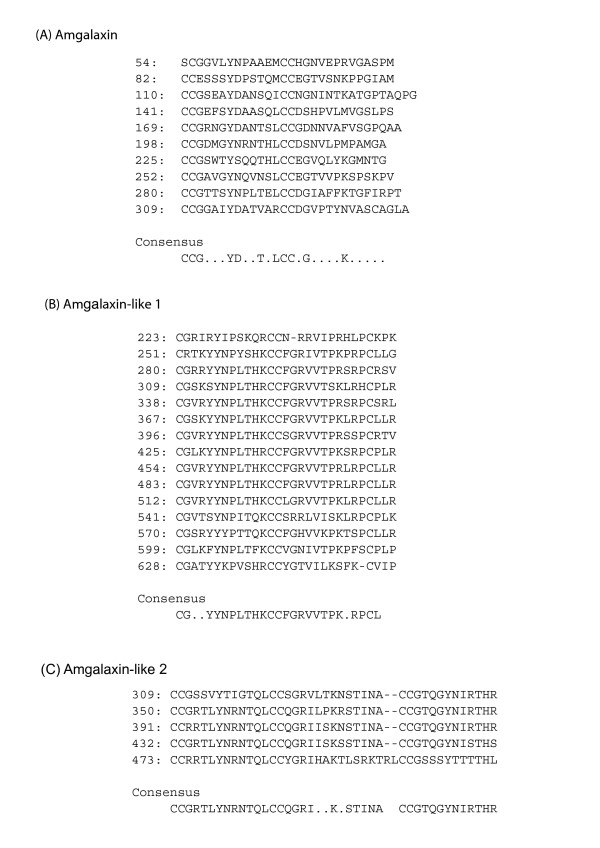
**Alignments of the Cys rich repeats of the three proteins**. Numbers on the left indicate the position in the predicted protein. A consensus sequence for the repeats from each protein is shown beneath the alignments. Residues are included in the consensus if they are represented in at least 50% of the repeats.

The *Acropora *and *Galaxea *galaxins are very similar in most structural characteristics. Like its *Galaxea *counterpart, Amgalaxin is cysteine-rich (12.4% overall, compared to 13.4% in Gfgalaxin). The 338 AA *Acropora *predicted protein (Figure 2A) consists of an N-terminal signal peptide of 23 AA, the bulk of the rest of the protein consisting of tandem repeats, each of around 30 AA and containing two di-Cysteine motifs (Figure 3A). Both proteins have a dibasic recognition site for processing endoproteases giving predicted mature proteins of 298 amino acids (Gfgalaxin) and 292 amino acids (Amgalaxin). The proteins are also similar in terms of predicted isoelectric points; for the mature proteins, pI values of 4.52 (Amgalaxin) and 4.53 (Gfgalaxin) were calculated. Gfgalaxin is a glycoprotein [8]; however, only one of the two potential N-glycosylation (i.e. N-X-S/T motif) sites identified in Gfgalaxin is conserved in the *Acropora *protein (Asn178). The high content of threonine and serine residues (14% and 8.9% in Gfgalaxin and Amgalaxin, respectively) suggests that O-glycosylation may be more significant. However, NetOGly  predicts only two positions with significant likelihood of O-glycosylation in Amgalaxin and a single potential site in Gfgalaxin.

### The Amgalaxin-like proteins are related but divergent

The cDNAs encoding both of the Amgalaxin-like proteins are larger than that encoding Amgalaxin, as are the predicted proteins themselves; Amgalaxin-like 1 is predicted to be 660 AAs and Amgalaxin-like 2 is 582 AAs. The Amgalaxin-like precursor proteins have a common domain structure that differs from the galaxins by the presence of a domain rich in acidic amino acid residues (Figure 2B and 2C). As in the case of the galaxins, signal peptides and Cys-rich repeat regions are present. Although the repeat units in the Cys-rich domain of Amgalaxin-like 1 are approximately the same size (29 AAs) as those in the galaxins, one difference that is potentially significant with respect to protein folding and higher order structure is the spacing of the cysteine residues. Whereas the galaxin proteins have two di-Cys motifs per repeat unit, Amgalaxin-like 1 has only one. The other two cysteine residues in each repeat are separated by three amino acids. The basic repeat unit in Amgalaxin-like 2 is 41 amino acids long and contains three di-Cys motifs. As in the case of Amgalaxin-like 1, the repeats in Amgalaxin-like 2 are more similar to each other than are the Amgalaxin repeats. When the consensus sequences of the Cys-rich repeats of the three proteins are compared it is apparent that Amgalaxin-like 2 and Amgalaxin share the greatest similarity (Figure 3). Although consensus sequences can be constructed for the galaxin and Amgalaxin-like repeats there is little similarity between the proteins apart from the arrangement of the dicysteine residues.

Amgalaxin and Amgalaxin-like 2 are both predicted to be moderately acidic proteins (pIs of 4.52 and 4.6 respectively for the mature proteins), whereas the mature Amgalaxin-like 1 protein is more basic (predicted pI of 9.09). The mature Amgalaxin-like proteins contain 13% and 16% Aspartic (Asp) and Glutamic (Glu) acid residues, predominantly located in one part of the protein, the acidic domain (Figure 2B and 2C).

NetNGlyc  predicts N-glycosylation at Asn48 in Amgalaxin-like 1 with high probability, but does not predict glycosylation at the single N-X-S/T site in Amgalaxin nor at either of the potential sites in the *Galaxea *protein. NetOGlyc  does not predict any likely O-glycosylation sites in Amgalaxin-like 1. The mature Amgalaxin-like 2 protein contains three potential N-glycosylation sites and several potential sites for O-linked glycosylation.

### Expression analysis of *A. millepora galaxin*-related genes

**Figure 4:**
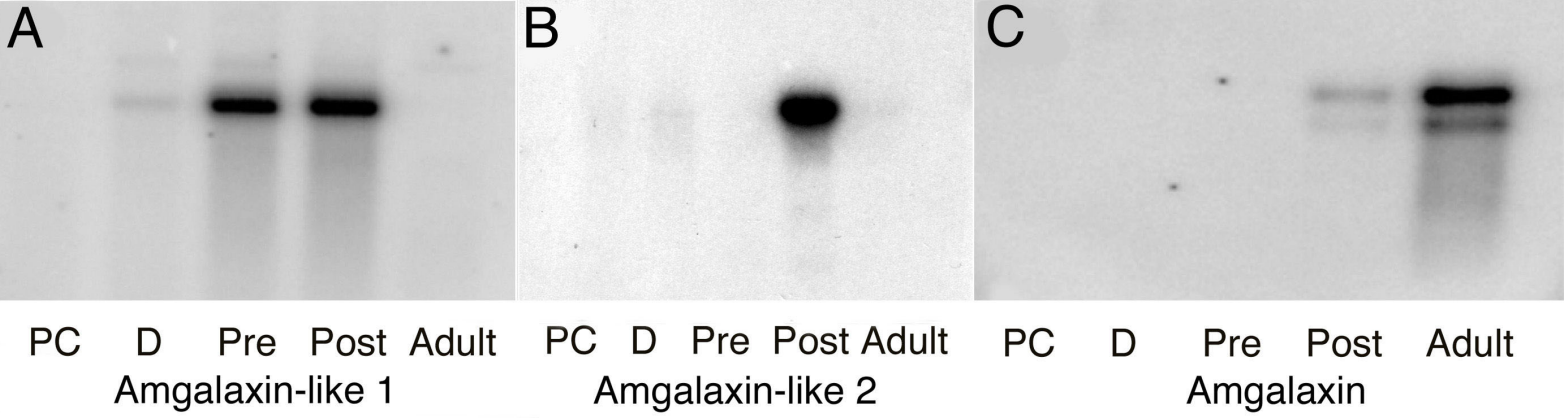
**Virtual northern blots of the three genes arranged in the order in which they are expressed**. The various stages represented on each blot are labelled across the bottom of the blot. *Amgalaxin-like 1 *expression first appears weakly even before gastrulation and is strong both before and after settlement, but is not apparent in the adult. *Amgalaxin-like 2 *is expressed exclusively immediately after settlement. *Amgalaxin *apparently has two isoforms both of which are expressed weakly immediately after settlement and more strongly in the adult. Abbreviations: PC = prawn chip, D = donut, Pre = presettlement planula larva, Post = polyp immediately after settlement.

Virtual northern analyses show that the *Amgalaxin *and *Amgalaxin-like *genes each have distinct temporal expression patterns (Figure 4). We will therefore consider these genes in the order in which they are expressed. *Amgalaxin-like 1 *is expressed strongly in pre-settlement planulae and primary polyps, but the mRNA was not detected in adult colonies (Figure 4A). *Amgalaxin-like 2 *expression is restricted to post-settlement polyps (Figure 4B). By contrast, as in the case of its *Galaxea *counterpart [8], *Amgalaxin *is expressed in both of the post-metamorphosis stages examined: weakly in primary polyps, but strongly in adult colonies (Figure 4C). In the case of *Amgalaxin*, two transcripts were detected on virtual northern blots (Figure 4C). The strong upper band, with an apparent size of approximately 2 Kb, corresponds to the sequence shown; the lower (~1.7 Kb) band is assumed to correspond to an alternatively spliced product.

**Figure 5:**
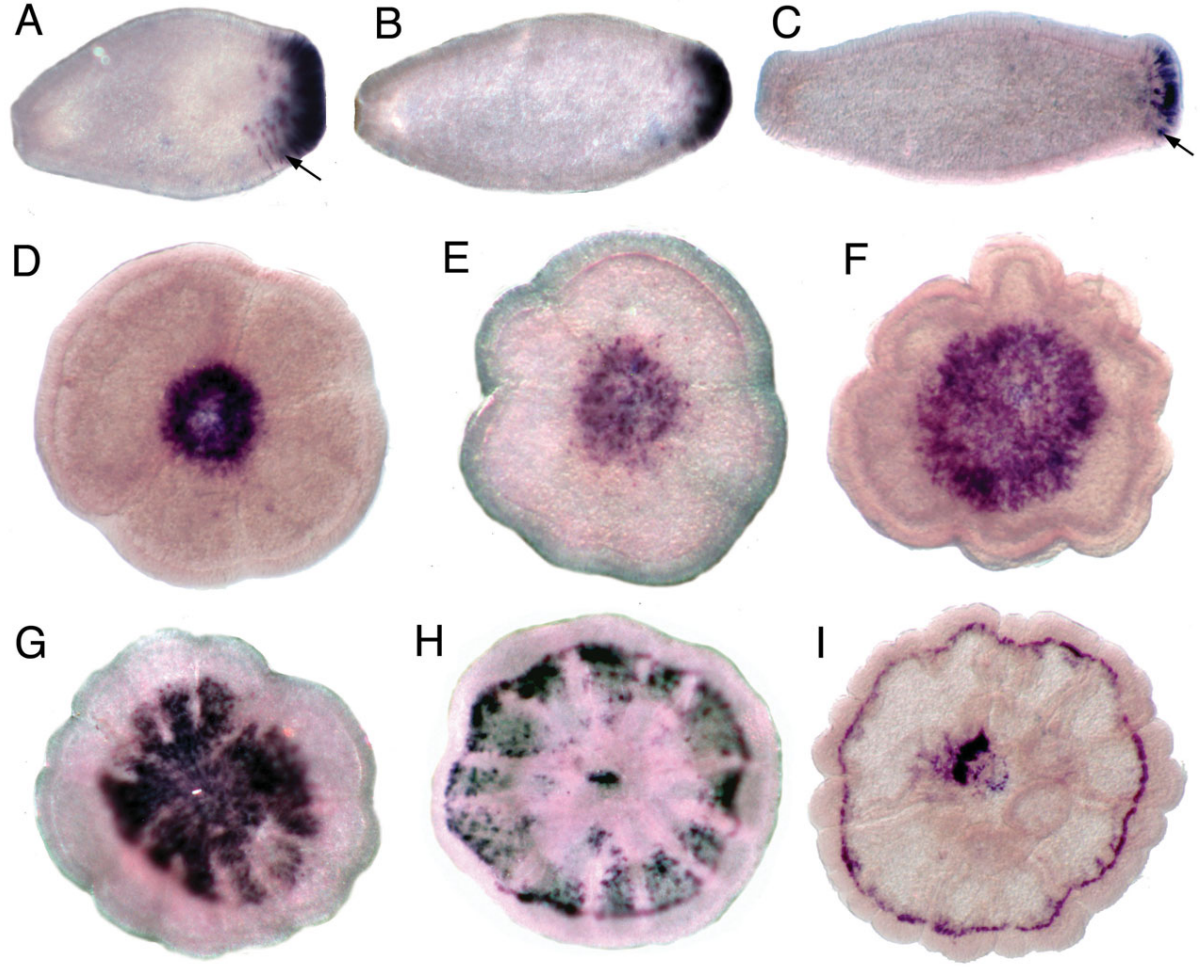
**Developmental expression of *Amgalaxin-like 1***. (A-C) Expression begins in a zone of strong expression at the aboral end of the planula. At the margins of this zone the expressing cells are no longer contiguous (arrows). (D-F) After settlement the zone of expression on the aboral side of the polyp expands as the polyp ages. (G-H) The zone of expression then begins to fragment segmentally, eventually leaving a few traces of expression centrally as well as a submarginal ring of expression (I). Planulae are shown in lateral view with the aboral end to the right. (D-I) are aboral views.

*Amgalaxin-like 1 *spatial expression is first detected in a subset of aboral ectodermal cells of the mid-late planula (Figure 5A–C, arrows; see Figure 1A and 1B), During settlement and metamorphosis, *Amgalaxin-like 1 *expression remains restricted to this ectodermal cell population (Figure 5D–I; Figure 1B). At this time, changes in the overall morphology of the larva can be observed as it flattens down onto the substratum and mesenteries are formed. After the larva has flattened (Figure 5D, Figure 1C), the *Amgalaxin-like 1 *expression domain expands radially from the former aboral end of the planula to near the edge of the now flat primary polyp (Figure 5H–I). This outer, sub-marginal ring of expression on the aboral side persists until about the 12-septum stage (Figure 1D, E) before gradually dissipating.

**Figure 6:**
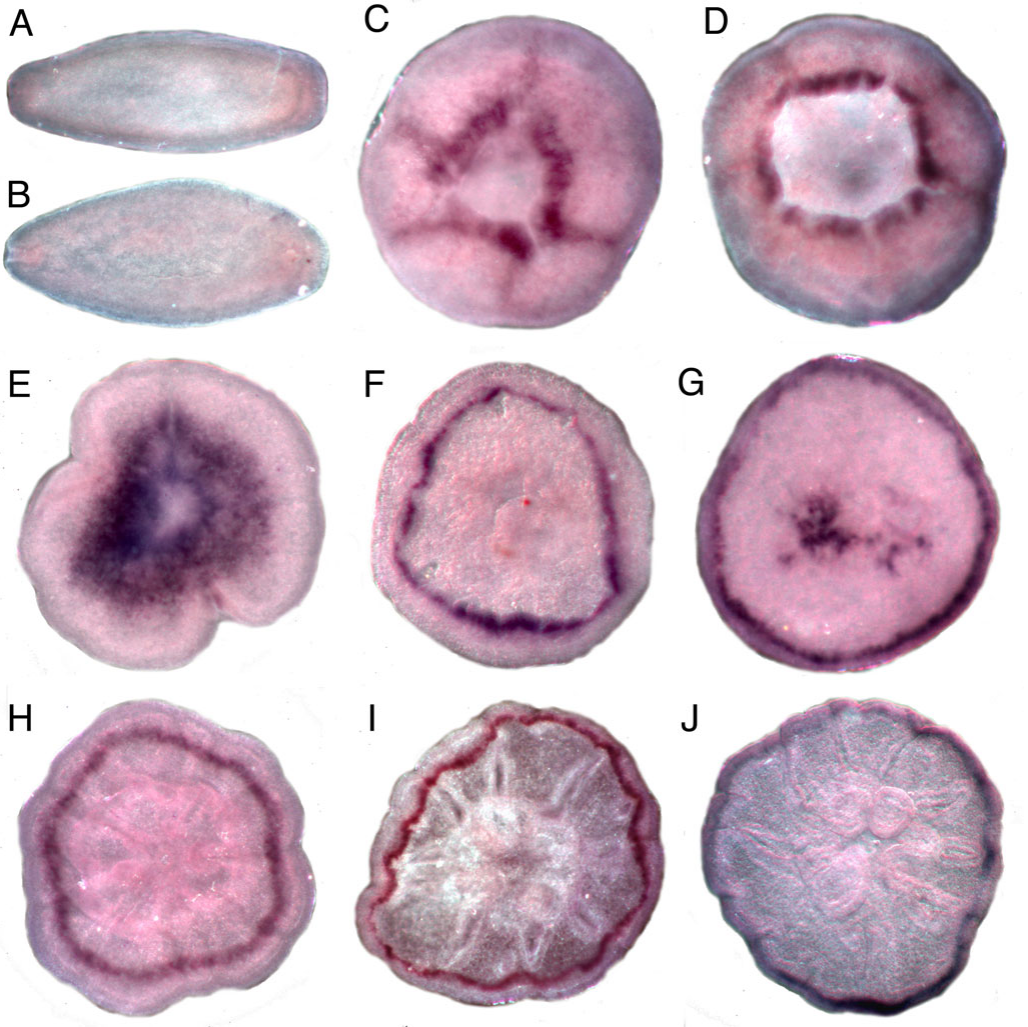
**Developmental expression of *Amgalaxin-like 2***. There is no expression in the planula larva (A-B). Expression first appears as a ring on the aboral side of the planula as it shortens to form a sphere (C-D). Expression continues aborally, sometimes in a zone (E) and sometimes in a submarginal ring (F-J) as the polyp ages. In (F)(H) and (J) the polyp is viewed orally and the aboral expression is seen through the cleared tissue.

Consistent with the developmental virtual northern blot (Figure 4B), *Amgalaxin-like 2 *expression is not present in the pre-settlement planula larva (Figure 6A, B), first appearing as the spindle-shaped planula shortens into a sphere (Figure 6C, D) before flattening against the substratum (Figure 6E–J; Figure 1C). Thereafter, expression is localized to an expanding ring in the aboral ectoderm (Figure 6C–J), in its final stages (Figure 6H–J) resembling the expression of *Amgalaxin-like 1 *(Figure 5I).

**Figure 7:**
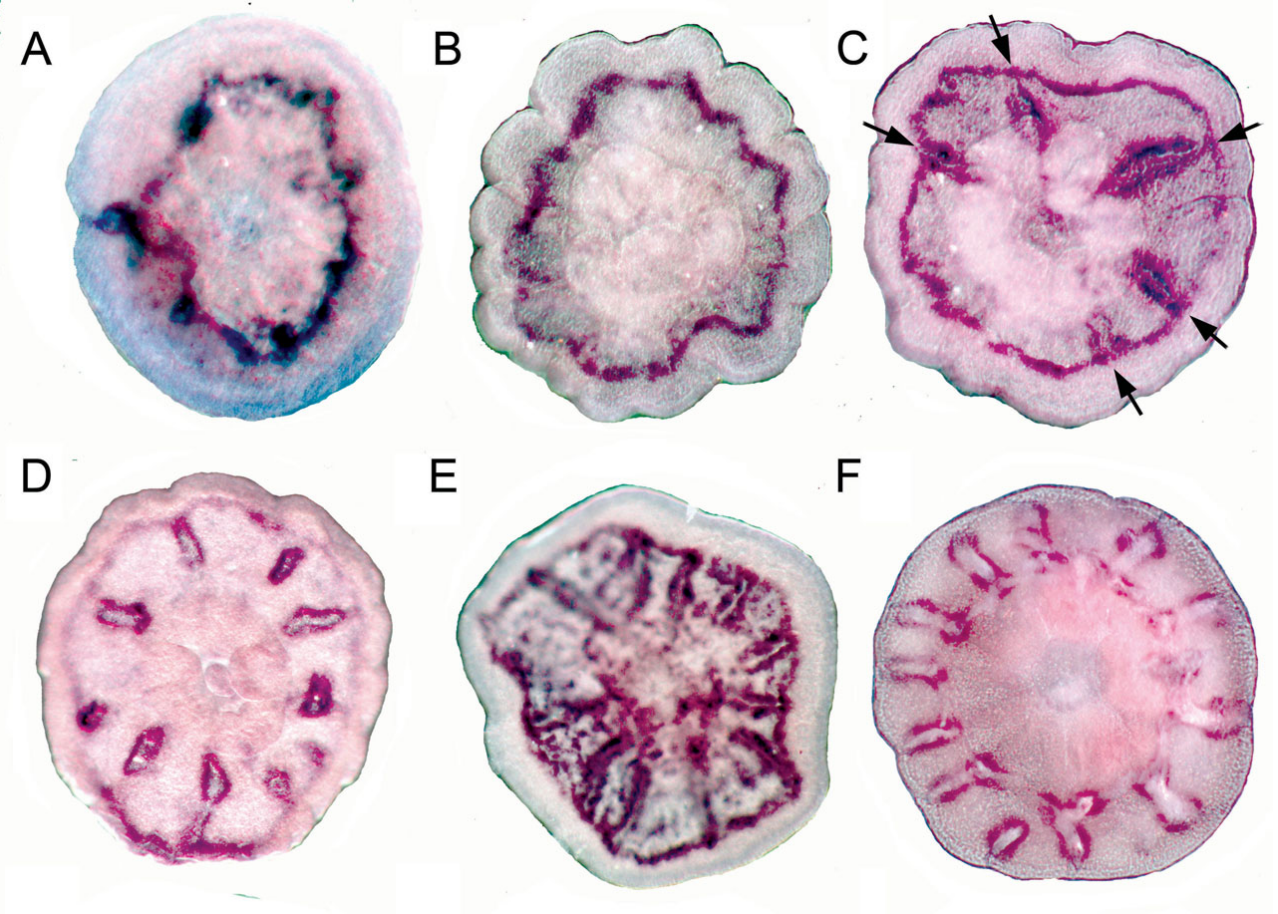
**Developmental expression of *Amgalaxin***. Expression begins as an aboral submarginal ring (A-B) Septal expression is then added (arrows) as the polyp grows older (C-F). There is also sometimes aboral granular expression between the septa (E). Septal expression continues in the oldest polyp studied (F). (A), (C) and (E) are viewed aborally, (B), (D) and (F) are viewed orally.

Although virtual northern blot analysis showed that *Amgalaxin *expression was much stronger in the adult, expression was also present in immediate post-settlement stages. This finding is consistent with in situ hybridization results, which show expression beginning in an aboral ring resembling those seen for *Amgalaxin-like 1 *and *2 *(Figure 7A). However, there is one marked difference between *Amgalaxin *and the *galaxin-like *transcripts in that *Amgalaxin *is expressed along the calcifying septa from shortly after their initiation (Figure 7C–F; Figure 1D) and was still apparent there at the twelve septum stage in the oldest polyp which we attempted to stain (Figure 7F).

## Discussion

All three of the *Acropora *galaxin-related proteins contain cysteine-rich domains including tandemly repeating di-Cys motifs (Figures 2, 3). Although the consensus repeat sequences have some similarities, evolutionary relationships between the three proteins are unclear at this stage. Phylogenetic analyses of such divergent proteins would be inappropriate, and to resolve their evolutionary relationships will require data from a phylogenetically representative range of corals, including basal clades. The presence of the Cys-rich domains is consistent with these proteins having a structural role in the organic matrix, as framework proteins are frequently Cys-rich [14,23] and tandem repeats with conserved Cys residues are commonly found in many types of extracellular matrix and cell surface receptor proteins [23,24]. Double Cys motifs may form intramolecular cross-links via disulfide bonds [23]. However, in vitro studies with synthetic peptides indicate that dicysteine motifs may efficiently form cyclo-cystine loops [25], limiting their ability to form intra- (or inter-) molecular cross links [11]. Fukuda et al. [8] suggest that therefore only the two terminal Cys residues may be available for intermolecular disulfide bonds.

Although only distantly related to the scleractinian galaxins, proteins with similar properties have been identified as components of the organic matrix of molluscs. For example, di-Cys motifs are found in lustrin A from *Haliotis rufescens *[23], the N14 protein from *Pinctada maxima *[26] and in pearlin from *Pinctada fucata *[27]. These similarities raise the possibility of common functions in biomineralization [8,11], either in establishing an overall structural framework onto which other matrix components may be deposited or in actively controlling crystal nucleation and development.

### The Amgalaxin-like proteins combine characteristics of two distinct protein families involved in mollusc calcification

Although Amgalaxin-like 1, Amgalaxin-like 2 and Amgalaxin are clearly structurally related, one major difference is the presence of an acidic domain in the galaxin-like molecules that is absent from both the *Acropora *and *Galaxea *galaxin proteins. Proteins rich in acidic amino acid residues are important components of the organic matrices of a number of taxonomically diverse calcifying invertebrates. For example, Gotliv *et al.*, [14] identified a family of Asp-rich proteins associated with the mineral component of the bivalve *Atrina rigida *and suggested roles in controlling mineralization. Ameye *et al.*, [28] identified an acidic N-glycoprotein in the organic matrix of spicules from the sea urchin *Paracentrotus lividus *and Rahman *et al. *[29-31] found acidic proteins that bind calcium in the organic matrix of spicules of the alcyonarians *Sinularia polydactyla *and *Lobophytum crassum *and reported that both species possess aspartic acid rich matrices. Although there are no direct precedents from scleractinians, Puverel *et al. *[32] found that matrices of both *Stylophora pistillata *and *Pavona cactus *were high in acidic amino acids (45.9 and 65.5% acidic amino acids, respectively) and obtained an internal sequence from *S. pistillata *that contained a long (36 residue) poly-Asp domain.

While the overall pI value for Amgalaxin-like 1 mature protein is 9.09, charged amino acid residues are distributed in a non-uniform way: the values for the acidic and Cys-rich regions are 3.21 and 12.39, respectively, and the Amgalaxin-like 2 protein has a similar bipartite structure. The pIs of the acidic domains are in the same range as those reported for some acidic protein components of molluscan matrices [14], while the pIs of the Cys-rich regions resemble those of a family of matrix proteins from the pearl oyster *Pinctada fucada *(pI values of 9.5 and 9.8; [33]).

The Amgalaxin-like proteins therefore combine characteristics that in molluscs reside in two distinct families of matrix proteins; an Asp-rich family [14], which might actively control mineralization [8], and a family of basic proteins that could be involved in linking hydrophilic molecules to the framework of the organic matrix, or binding carbonate [33].

### What is the function of the Amgalaxin-like proteins?

Although the galaxin-like proteins have an overall structural similarity, they are otherwise dissimilar at the primary amino sequence level, and may be expected to fulfill somewhat different functions during calcification. Their sequential expression during development (Figure 4) is consistent with this suggestion.

Deposition of the coral skeleton is thought to occur as a two-step process; the first step is the formation of early mineralization zones characterized by the presence of randomly oriented grains of CaCO_3_, followed by a second step characterized by crystal-like fibers of aragonite. This pattern of mineralization has been described in newly settled *Pocillopora damicornis *[19] and *Porites porites *[34] and in axial polyps of *Acropora cervicornis *[35]. Although these two kinds of calcification probably both occur throughout the coral life cycle [3], several lines of evidence suggest that granular calcification predominates during early coral development, whereas calcification may be largely of the fibrous type in adult colonies. However, it is only in newly settled polyps that the two processes can be studied in isolation.

Goreau and Hayes [34] describe the beginnings of skeleton formation as the secretion of "mucoid substances" which serve to cement the settling planula to the substratum and it is possible that this could be the function of the Amgalaxin-like 1 protein. In contrast, in their descriptions of the early post-settlement stages Vandermeulen and Watabe [19] and Le Tissier [16] concentrated on the calcified elements of the early skeleton, perhaps because of the preparation techniques that they used. The earliest post settlement stage of *Pocillopora *shown by Le Tissier ([16], Figure 2a) has a complete circular rim of calcified material on the basal plate with scattered areas of calcification within this circle, while in *Porites *Goreau and Hayes ([34], Figure 4-2) show a portion of the rim of the plate as calcified with scattered areas of calcification on the plate. Clode and Marshall [17] measured intracellular calcium in pre- and post-settlement larvae of *Pocillopora damicornis *using Calcium Orange fluorescence and found that levels of intracellular calcium were lowest in a small area at the aboral end of pre-settlement and immediate post-settlement planulae. This area corresponds to areas of *Amgalaxin-like 1 *expression shown in Figure 5A–E. Whether there is a functional relationship between the two patterns remains to be investigated. The expression of *Amgalaxin-like 2*, in contrast, essentially demarcates the outer limits of the low Ca^2+ ^area seen in *Pocillopora*. So, the observed distribution patterns of the two Amgalaxin-like proteins are consistent with a role for Amgalaxin-like 1 in laying down the organic matrix in advance of calcification and for Amgalaxin-like 2 in the process of calcification itself. Another possibility is that transiently expressed proteins such as the Amgalaxin-like molecules may actually be inhibiting the formation of aragonite fibers in the basal plate while inducing calcite deposition. Indeed, there is an invertebrate precedent for such an action of acidic proteins since Addadi and Weiner [36] established that they could alter the morphology of calcite crystals by adding an acidic matrix protein from the bivalve mollusc *Mytilus*. It remains to be seen if the galaxin-like proteins have similar properties.

### Amgalaxin and the mesenteries: fiber-like calcification

Following its initial expression in an aboral ring resembling those seen for the *Amgalaxin-like *transcripts (Figure 7A–B), *Amgalaxin *is expressed along the calcifying septa in a pattern distinct from the *Amgalaxin-like *transcripts. The close correspondence between the appearance of mesenteries (and the implied initiation of septal mineralization) and the initiation of *Amgalaxin *expression is consistent with involvement of Amgalaxin-like in deposition of the basal plate and protosepta, Amgalaxin could thus be involved in the fiber-like calcification characteristic of mesenteries. The strong expression of *Amgalaxin *in adult *Acropora *and the abundance of the corresponding protein in mature *Galaxea *colonies [8] imply that galaxins may be involved in controlling the fiber-like aragonite deposition characteristic of adult skeletons. According to Vandermeulen and Watabe [19] the basal plate of *Pocillopora *contains a mixture of calcite and aragonite, so neither the circular expression pattern nor septal expression is inconsistent with the idea that the Amgalaxin-like molecules are associated with granular calcification and Amgalaxin with aragonite. Aragonite deposition greatly predominates in the adult coral and only Amgalaxin expression persists into that stage.

### Galaxins as examples of functional convergence after secondary recruitment?

Massive calcification is a widespread trait across the animal kingdom; obvious examples are found among echinoderms, molluscs, vertebrates and corals. Many lines of evidence indicate that the trait has evolved independently in these lineages despite the involvement of some common classes of molecules (e.g. carbonic anhydrases; [37]). Although the coral galaxins and the mollusc lustrin A/N14/pearlin proteins may have separate evolutionary origins, their structural similarity suggests common function. As in the case of mucoperlin, a component of the mollusc organic matrix [38], galaxins resemble mucins in that they have a high serine content, with consequent glycosylation potential, and have a tandem repeat structure. This structure is consistent with the idea that these and other ECM genes may have evolved from a mucin-type ancestor [39] and may have calcium binding activity. However, the experiments of Fukuda et al. [8] indicate that Gfgalaxin does not bind Ca^2+^. The galaxin-like proteins, with their two distinct domains, may combine the functions of two distinct classes of mollusc proteins – the acidic proteins that are thought to actively control mineralisation, and basic proteins that either bind bicarbonate or form links with the organic matrix. These hypothetical functions require future testing in vitro.

Although proteins containing multiple di-Cys motifs are widely distributed, clear orthologs of galaxin are so far known only from scleractinians. However, there are predicted proteins in the non-calcifying cnidarians *Nematostella *(e.g. EDO38853.1) and *Hydra *(XP_002169304; "usherin-like") having moderate degrees of similarity to the scleractinian galaxins, and a "galaxin" has been reported in the vestimentiferan tubeworm *Riftia pachyptila *[40]. There are also database entries annotated as either "similar to galaxin" or "galaxin-related" from *Ciona *and *Oikopleura *(AAS21342), respectively. The product of the *Ciona *("similar to galaxin") gene ci0100148033 gives a BlastP score of 2e^-31 ^against Amgalaxin (for comparison, BlastP similarity between Amgalaxin and Amgalaxin-like 1 is 4e^-29^) and the gene is expressed throughout the epidermis at the late tailbud stage (Aniseed database: ), while the *Riftia *"galaxin" was identified as body-wall specific in differential display experiments [40]. Together with the *Acropora *data, these various lines of evidence suggest that the ancestral galaxins may have been structural ECM proteins that were secondarily recruited to roles in skeletogenesis during the Triassic, when scleractinians first appear in the fossil record. Comparison of sequences and expression data across a broad range of cnidarians and other animals will be required to clarify the evolution of this protein family.

## Conclusion

The distribution of the transcripts reported here, in addition to the information available from other sources, indicate that proteins containing di-Cys repeats are frequently involved in the formation of organic skeletons and may have evolved from structural ECM proteins. Such knowledge is of particular importance at this time due to concern over the effects of ocean acidification on the ability of marine organisms to calcify.

## Methods

### Fixation and storage

*A. millepora *embryos and larvae were fixed for 15–60 min in 3.7% formaldehyde in Millipore-filtered seawater (MPFSW) buffered to pH8.0 with Hepes buffer. Fixed material was washed repeatedly in MPFSW, dehydrated through a graded methanol series and stored in absolute methanol at -20°C until needed.

### In situ hybridization and image capture

Riboprobe synthesis and in situ hybridization were performed as reported by [41]. Following development embryos were washed in PBT and gradually dehydrated to 70% glycerol in which they were stored. They were mounted on microscope slides in 90% glycerol for photography. Digital images were captured with a Spot Camera mounted on a Wild Photomakroskop. Micrographs of embryos of varying stages were moved to a common white background and color and contrast adjusted for better visualization of staining patterns.

### Sequencing

DNA sequencing was performed using Big Dye Terminator v. 3.1 (Applied BioSystems) with vector and internal primers. Reactions were run on an ABI 3730 sequencer at the Biomolecular Resource Facility (JCSMR, ANU). Sequence anaysis was carried out using MacVector 9.5.2 (Accelrys) and Lasergene (DNASTAR).

### RACE

A 520 bp Amgalaxin-like 2 sequence was identified in a screen for genes which are differentially expressed during metamorphosis (unpublished). 5' and 3' RACE were carried out with primary polyp RNA following the Clontech SMART RACE cDNA Amplification kit procedure. The primers used were: 5'RACE: 5'GATGGTGCTGTTTTTGGTGAGGAC3'; 3'RACE: 5'CTGCTGTAGTGGTCGTGTCCTCAC3'. The PCR products were ligated into pGEM-T Easy (Promega). The predicted cDNA sequence was assembled from the overlapping 5' and 3' sequences using SeqMan (DNASTAR). The existence of the predicted transcript was confirmed by using the following 5' UTR and 3' UTR primers to generate a product of the predicted size and sequence: 5'UTRprimer: 5'AACAGAGGAGATAGCTAGTGT3'; 3'UTRprimer:5'GTCCTCCTCGAGGTTACATCAC3'.

### Virtual northern blots

"Virtual northern blots" were made using the Clontech SMART cDNA Synthesis Kit, according to the manufacturer's instructions using RNA from the stages indicated.

## Authors' contributions

All authors contributed to all aspects of the paper, with AR and ZL making major contributions on data collection while DM, DH and EB focussed on writing and data presentation.
